# Development, Characterization, and Antibacterial Analysis of the Selenium-Doped Bio-Glass-Collagen-Gelatin Composite Scaffold for Guided Bone Regeneration

**DOI:** 10.7759/cureus.48838

**Published:** 2023-11-15

**Authors:** Sushma B Reddy, Parkavi Arumugam, Obuli G Kishore, Saranya K

**Affiliations:** 1 Periodontics, Saveetha Dental College and Hospitals, Saveetha Institute of Medical and Technical Sciences, Saveetha University, Chennai, IND

**Keywords:** innovations and health, quality of life, bioglass, selenium, bioactivity, guided bone regeneration

## Abstract

Background

Guided bone regeneration (GBR) is an often-used technique to aid the successful placement of dental implants in sites with deficient bone. The search for the ideal GBR membrane with bioactive components improving the regenerative outcomes is still on. In this study, a novel composite GBR membrane was developed using selenium-doped bio-glass, collagen, and gelatin. It was further characterized for surface, chemical, biocompatibility, and antibacterial properties.

Methodology

Selenium-doped bio-glass was prepared using the sol-gel method. The membrane was fabricated using an equal ratio of collagen and gelatin mixed with 1% selenium-doped bio-glass. The solution was poured to obtain a thin layer of the material which was lyophilized to obtain the final GBR membrane. The membrane was analyzed with scanning electron microscopy, energy dispersive X-ray (EDX) analysis, attenuated total reflectance-Fourier transform infrared spectroscopy (ATR-FTIR), zebrafish cytotoxicity test, and antibacterial assay.

Results

The membrane revealed good surface roughness with lamellar and fibrillar arrangement with a minute granular surface ideal for cell attachment and proliferation. The EDX analysis revealed the presence of carbon, oxygen, and nitrogen as predominant components with trace amounts of calcium, phosphorus, silica, and selenium. Fourier transform infrared spectroscopy analysis also proved the presence of collagen, gelatin, and bio-glass. The membrane revealed excellent biocompatibility with zebrafish growth at a normal rate with 90% viability maintained at 48, 72, and 96 hours and 95% viability at 120 hours. It also exhibited excellent antibacterial activity against *Staphylococcus aureus *and *Escherichia coli* with minimal growth of bacterial colonies.

Conclusion

The developed novel selenium bio-glass collagen and gelatin composite scaffold has a good surface and antibacterial properties along with excellent biocompatibility. Further cell line and in vivo studies should be conducted to explore its role in bone regeneration.

## Introduction

In today's modern era, edentulism is a major cause of concern among the aging global population. According to a recent global analysis of the disease burden of edentulism conducted in 2019, the number of prevalent cases and disability-adjusted life years of edentulism were 35.2 million and 9.6 million, respectively [1]. With patients craving functionally and aesthetically stable tooth replacements, implants are the sought-out contemporary treatment of choice. However, inadequate quantity and poor quality of bone, limit the utilization of implant-based prosthesis [2].

Guided bone regeneration (GBR) is a potential solution for sites with inadequate bone volume, where vertical and horizontal augmentation of the defect site for implant placement is possible. Studies have reported similar survival rates for implants placed in augmented sites when compared to implants placed in pristine sites [3,4]. The technique employs GBR membranes along with bone grafts for the regeneration of the lost tissues. The goal of membrane application is to attain defect isolation while maintaining the space for new bone formation [5,6]. An ideal GBR membrane should satisfy the properties of space maintenance, occlusive functionality, biocompatibility, bioactivity, and easy manipulation [7]. Though the technique has great promise, complete and predictable regeneration of the lost bone is still a complex process [8]. Soft tissue dehiscence leading to defect site exposure and subsequent contamination of the GBR membrane is the main complication associated with the technique [9,10].

Currently, various generations of GBR membranes have been developed and are in use. The first-generation GBR membranes are non-resorbable membranes that are associated with increased morbidity due to the need for a second surgery. Second-generation membranes are resorbable membranes that are associated with poor mechanical strength and unpredictable rate of degradation which affect the regenerative process [11]. Third-generation membranes have been developed with the incorporation of bioactive agents that aid in predictable tissue regeneration. A bioactive composite scaffold is a combination of natural and synthetic components with greater compositional similarity to native tissue and better mechanical and biological properties.

Of the various natural substances, collagen, and gelatin are the most favored biomaterial [12]. Collagen is a ubiquitous structural protein found in the mammalian extracellular matrix that has the unique property of being soft and flexible with greater tensile strength. It has greater compositional, structural, and functional similarity to the extracellular matrix of the native tissue. Gelatin is the denatured form of collagen that exhibits good hydrophilicity and gelation behavior. Both collagen and gelatin provide biomimetic cues for appropriate cell differentiation, and proliferation [13] while exhibiting good bio-affinity, biodegradability, and excellent biocompatibility. However, collagen lacks adequate mechanical stability and has a varied degradation rate that is modified by factors such as the origin, molecular weight, and the tissue characteristics of the source tissue [14]. These drawbacks of collagen membrane can be overcome by the addition of gelatin which greatly improves the handling characteristics of the composite membrane thereby improving the maneuverability.

Components that improve osteogenesis and osteo-induction like bone grafts, enamel matrix proteins, and bone morphogenetic protein are also being explored. Bio-glass is a bioactive form of silicon-based glass ceramics composed of silicon dioxide, sodium oxide, calcium oxide, and phosphorus pentoxide with a three-dimensional silicon dioxide network. In an aqueous environment, the highly reactive surface causes the hydrolysis of the silica matrix to form ortho-silicic acid and silanol leading to an increase in the pH levels. This further leads to hydrolytic degradation and the formation of hydroxyapatite layers on the surface. Due to its bioactivity, it forms a firm bond with the surrounding bone which further improves the osteo-induction and osteogenesis [15].

To enhance the biologic properties of the bio-glass various noble metal nanoparticles have been doped on it. Selenium is one such trace mineral of promise that shows proven antioxidant activity. Studies have now been exploring the antibacterial properties of selenium [16,17]. Selenium-doped bio-glass would have the advantage of antioxidant, anti-inflammatory, osteo-promotive, and antibacterial properties expressing a synergistic effect that would be ideal for regeneration. In an effort to progress towards predictable regeneration, a novel selenium-doped bio-glass-collagen-gelatin composite GBR membrane was developed. To the best of our knowledge, no other study has developed this GBR membrane. The membrane was further analyzed to assess its properties. The aim of the study was to develop and characterize the surface, chemical, antibacterial, and cytotoxic properties of the selenium-doped bio-glass-collagen-gelatin GBR membrane.

## Materials and methods

The study was conducted at the Department of Biomaterials at Saveetha Dental College. For the conduction of the developed selenium-doped bio-glass-collagen-gelatin composite scaffold's toxicity analysis with zebrafish, approval was obtained from the Institutional Ethical Committee for Animal Research, Saveetha Dental College (PROTOCOL NO: BRULAC/SDC/IAEC/SIMATS/05-2022/129).

Synthesis of selenium-doped bio-glass

All the chemicals and reagents of analytical grade were used. For the preparation of bio-glass, 45% tetraethyl orthosilicate in 10 ml of double-distilled water was mixed with 3 ml of nitric acid and allowed to gel over a duration of half a day. To this mixture, 24.5% calcium nitrate, 23.5% sodium nitrate, and 6% orthophosphoric acid were added and allowed to react. Each compound was dissolved separately. To this resultant mixture, 1% selenous acid was doped. The sol-gel reaction product was dried overnight and then sintered at 500℃ for one hour to obtain the selenium-doped bio-glass particulate graft.

Fabrication of collagen-gelatin-selenium bio-glass composite GBR membrane

The fish-origin collagen for the membrane preparation was sourced from Sisco Research Laboratories Chemicals India Pvt. Ltd. (SRL) (Mumbai, India), and gelatin was obtained from Merck Group (Darmstadt, Germany). For the fabrication of the GBR membrane, 15 ml of an equal volume of 10% collagen and 10% gelatin solution was mixed with 1% selenium-doped bio-glass. This solution was mixed to obtain a homogenous mixture that was poured on a petri dish to obtain an even-thin layer. The petri dish with the layer of the composite material was then lyophilized at -80℃ for 24 to 48 hours to obtain the final GBR membrane.

Material characterization

The composite GBR membrane was examined for surface properties using a scanning electron microscope (SEM) JSM IT800 (JEOL Ltd., Tokyo, Japan). The sample was coated with platinum sputtering for 30 seconds and then visualized under SEM using a 3kV accelerating voltage at 10 micro-meter resolution. Energy dispersive X-ray (EDX) analysis was done using the Oxford instrument to chemically characterize the sample. Vibrational modes were confirmed through Fourier transform infrared spectroscopy (FTIR) performed using Bruker ALPHA II (Bruker Corporation, Billerica, USA).

Toxicity analysis with zebrafish

The zebrafish model was used to analyze the in vitro toxicity of the synthesized GBR membrane. The stock suspension with the GBR membrane was used. About 25 numbers of zebrafish embryos were selected for the analysis. The zebrafish were well managed and assessed at different time periods. The number of dead zebrafish was estimated and differentiated with control samples. The zebrafish embryos were maintained at individual trails at a standard temperature for the generation of organs like the head, tail, and eyes. The embryos were observed under a microscope at 40x magnification every 24 hours. At every four-hour time period, the viable and dead embryos, and fishes were identified. The dead embryos and fishes were discarded at every inspection to restrict the contamination of the suspension. The mortality ratio was calculated for every 24 hours.

Antibacterial analysis

*Staphylococcus aureus* RB13U1 (MTCC 9542) and *Escherichia Coli *25922 (MTCC 443) bacterial species were used to analyze the antibacterial activity of the bioactive GBR membrane. The colony count method was used to estimate the antimicrobial activity of the membrane. Bacteria were inoculated individually with 20 ml of Luria-Bertani broth media. The loop of culture was introduced into sterile broth and incubated at 37℃ in an orbital shaker. After reaching the mid-log phase the cells were centrifuged at 4000 rpm for 15 minutes following which the pellets were obtained. The pellets were then washed thoroughly with phosphate-buffered saline. An experimental stock solution was prepared with the GBR membrane. The cells were used to analyze the antibacterial properties of the membrane using this solution. The experimental stock solution was incubated in the presence of the bacteria for six hours. Serial dilutions were used to smear the sterile nutrient agar plates with the GBR membrane stock solution. The plates were incubated overnight to analyze the growth of the colonies.

## Results

SEM analysis

**Figure 1:**
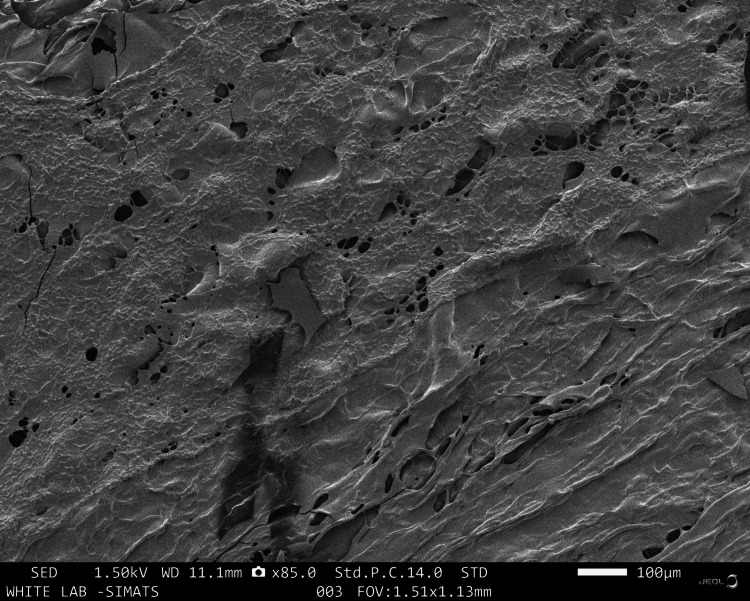
Scanning electron microscope (SEM) images of the selenium-doped bio-glass-collagen-gelatin GBR membrane GBR: Guided bone regeneration

The SEM analysis of the GBR membrane revealed minor surface roughness with minute surface porosities distributed sparsely. The rough surface also revealed a fibrous and lamellar structure that may be attributed to the collagen-gelatin components of the scaffold (Figure 1).

EDX analysis

**Figure 2:**
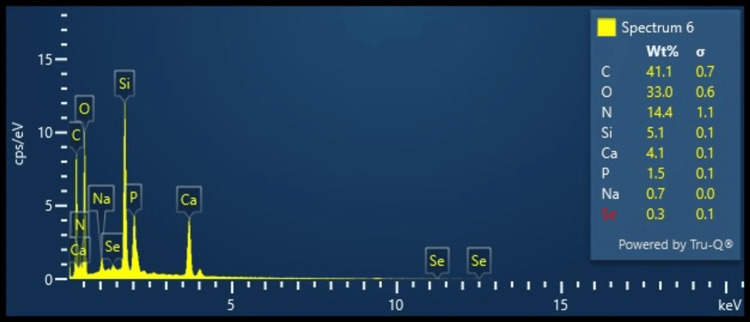
Energy dispersive X-ray (EDX) analysis of the developed GBR membrane GBR: Guided bone regeneration

EDX analysis revealed the presence of carbon, oxygen, and nitrogen as the predominant elements which can be attributed to the amide functional groups of collagen and gelatin. The presence of calcium, phosphorus, sodium, and silica can be attributed to the presence of bioactive glass. The presence of trace amounts of selenium proves the successful doping of selenium onto the bio-glass (Figure 2).

Infrared spectrum analysis

**Figure 3:**
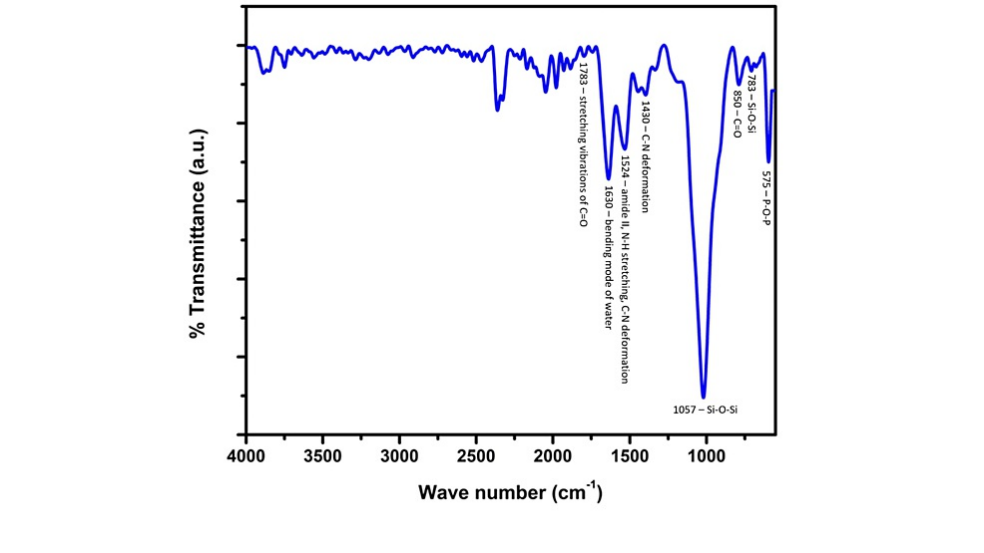
Fourier transform infrared spectroscopy (FTIR) analysis of the developed GBR membrane The results showed characteristic absorbance peaks corresponding to the presence of functional groups. Stretching vibration of C=O and the presence of the carbonate group corroborated the peak at 1783 and 850, respectively; the bending mode of water corroborated the peak at 1630; amide II, N-H, and C-N functional groups corroborated the peak at 1524; C-N deformation corroborated to the peak at 1430; stretching vibrations and deformation of Si-O-Si functional group corroborated to the peaks at 1057-783 and P-O groups corroborated to the peaks from 1321-595. GBR: Guided bone regeneration

FTIR analysis was done to analyze the molecular compounds present in the scaffold (Figure 3). The results showed the presence of peaks or absorbance bands corresponding to the presence of amide groups, carbonate groups, siloxane groups, phosphoryl groups, and the bending mode of water. The bands at 1524 and 1430 correspond to the presence of N-H stretching and C-N deformation that may be attributed to the presence of the amide II functional group. This proves the presence of collagen and gelatin as amino groups. Similarly, the peaks at 1783, 1057, and 1024-575 correspond to the stretching of C=O, Si-O-Si, and bending vibrations of P-O which may be attributed to the presence of carbonate, siloxane, and phosphoryl functional groups (Table 1). This proves the presence of selenium-doped bio-glass in the membrane.

**Table 1:** Absorbance band depicted by the developed GBR membrane GBR: Guided bone regeneration

Wave number	Bond type	Functional group
1783	C=O	Stretching vibration of C=O
1630	H-O-H	Bending mode of water
1524	N-H, C-N	Amide II, N-H stretching, C-N deformation
1430	C-N	C-N deformation
1321	P-O	Bending and symmetric stretching vibration of P-O
1057, 1024	Si-O-Si, P-O	Siloxane group, Phosphoryl group
850	C=O	Carbonate group
783, 736	Si-O-Si, P-O	Bending and symmetric stretching vibrations of P-O and Si-O-Si
595, 575	P-O, P-O-P	Bending and stretching vibration of P-O, P-O-P

Biocompatibility analysis using zebrafish toxicity

**Figure 4:**
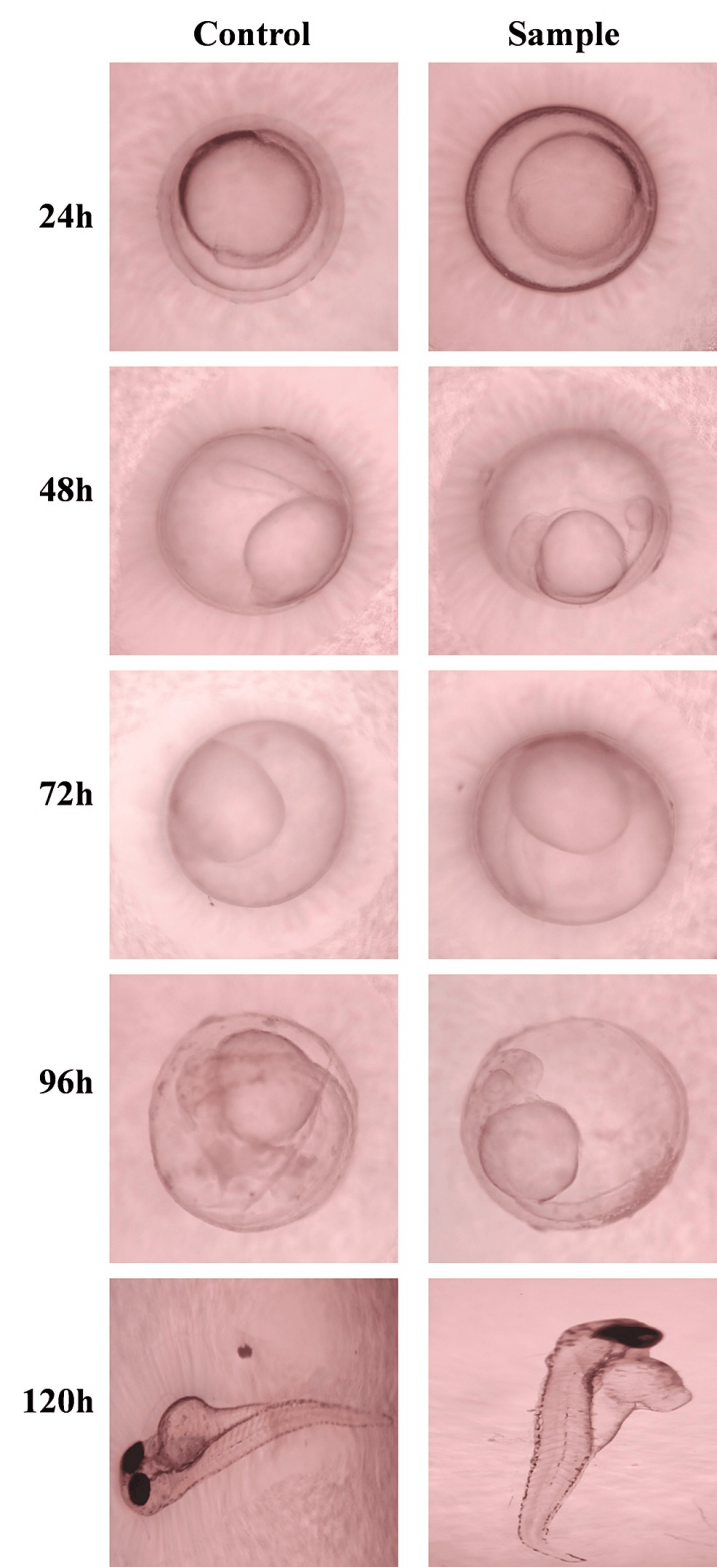
Images depicting the developmental stages of the test and control groups at various time periods

**Figure 5:**
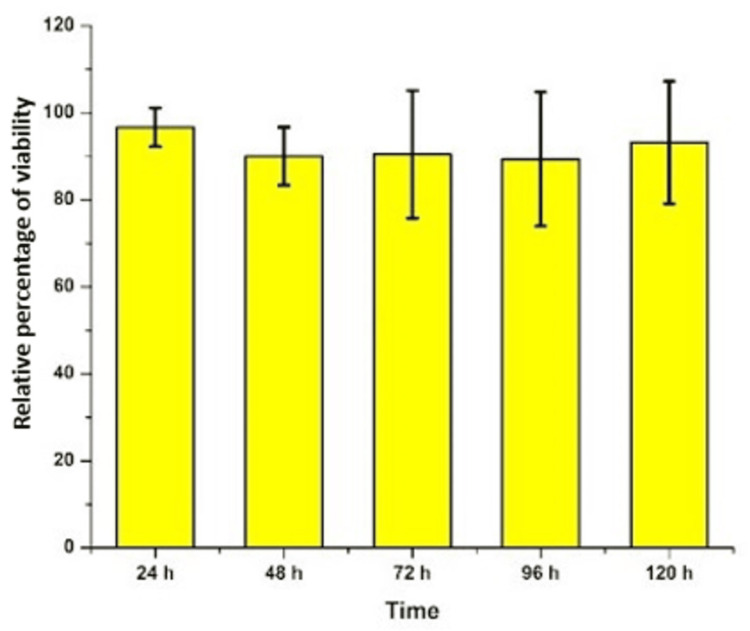
Relative percentage of viability of zebrafish

The zebrafish embryos were treated with the test and control extracts before the sphere stage (Figure 4). They were then periodically assessed for developmental abnormalities over 24-hour periods. There were no significant changes between the growth patterns noted in embryos treated with both test and control groups. At each 24-hour analysis, the development of the test group was at a similar rate to the development noted in the control group. At 48 hours both the groups showed the starting of the head and tail differentiation. At 96 hours and 120 hours, a well-developed embryo was observed in both groups. The relative percentage of zebrafish viability was maintained at 90% at 48, 72, and 96 hours. At 120 hours the relative viability was 95% (Figure 5). The results prove that the prepared GBR membrane extract had no toxic effects on the development of the zebrafish embryo.

Antibacterial assay

**Figure 6:**
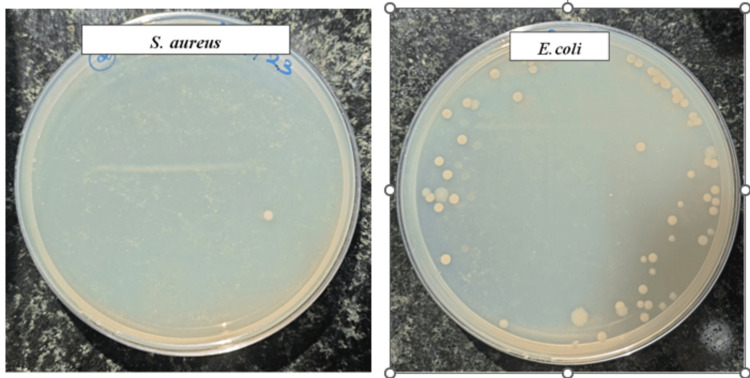
Antibacterial activity against common oral pathogens

The antibacterial assay was performed against *S. aureus* and* E. coli* using culture on nutrient agar. The GBR membrane-based extract had excellent antibacterial activity against *S. aureus *with almost no growth of bacterial colonies on the agar plate. A similar antibacterial effect was observed against *E. coli *with minimal growth of colonies on the agar plate. The results prove that the selenium-doped bio-glass-collagen-gelatin composite GBR membrane has excellent antibacterial activity against common oral pathogens and can be employed for predictable regenerative outcomes (Figure 6).

## Discussion

Based on tissue engineering principles, bioactive GBR membranes have emerged as the prospective solution for predictable bone augmentation. With a composite scaffold employing both bioactive and biocompatible components of both natural and synthetic origin, improved mechanical, osteo-inductive, and antibacterial properties have been instituted in the membranes. This would ultimately present an overall positive and predictable regenerative outcome. In the present study, a novel selenium-doped bio-glass-collagen-gelatin GBR membrane was developed and its properties were analyzed.

The prepared GBR membrane had good surface properties that are ideal for cell attachment and differentiation. The surface roughness due to lamellar structure and fibrillar arrangements could be attributed to the presence of collagen and gelatin in the membrane. Similar surface properties were observed in studies analyzing the collagen-based GBR membranes revealing marked differences in the microstructures with loosely arranged bundles of collagen [18]. As collagen is a ubiquitous compound present in the extracellular matrix of human tissues, it plays a significant role in biomimicry during the healing process leading to tissue regeneration. Both collagen and gelatin have excellent hydrophilicity, biocompatibility, and biodegradable properties. However, collagen membranes have been associated with poor handling properties. To improve the maneuverability of the membrane, gelatin was added as a component. Though gelatin is the denatured form of collagen, its addition has various advantages. The tertiary structure of gelatin enhances the mechanical features of the membrane while making it more versatile and highly adaptable. It also enables the incorporation of adhesive hydrogels that improve cell adherence, thus providing scope for further improvement of the membrane in the future. The surface also revealed a layer of minute granular surface that could be attributed to the presence of selenium-doped bioactive glass in the membrane. Though minute porosities were noted on the surface, they were not present through the cross-section of the membrane; thus, preserving the barrier function of the membrane. Also, the porosities observed were of less diameter than the size of the osteoblasts, fibroblast, and epithelial cells. Thus the minute surface porosities may act as the nodes for cell attachment and further proliferation.

The EDX and FTIR analysis further corroborated the presence of the compounds that were used for the synthesis of the membrane. With carbon, oxygen, and nitrogen as the predominant elements and calcium, phosphorus, sodium, and selenium as trace elements, the presence of collagen, gelatin, and selenium-doped bio-glass could be proved. In the FTIR analysis, the presence of the absorbance bands corresponded to the presence of amide functional groups. C-N deformation and H-N stretching prove the presence of collagen and gelatin. Similarly, the bending and stretching vibrations of Si-O-Si, C=O, and P-O depicted the presence of siloxane, carbonate, and phosphoryl groups which proved the presence of selenium doped bio-glass. Similar elements and absorption peaks were noted for a collagen-bio-glass-chitosan-based GBR membrane [19]. The release of the surface ions in an aqueous medium causes an acidic environment leading to the formation of apatite crystals bonding directly to the adjacent bone. This would act as nodes of ossification further improving the regenerative potential of the membrane. Moreover, the pH and osmotic changes also have been postulated to have an antibacterial effect at the defect site. Thus, the inclusion of bioactive glass would improve the osteo-inductive and antibacterial properties of the membrane.

The advantage of the addition of selenium was further analyzed with the antibacterial assay against common oral pathogens like *S. aureus* and *E. coli*. With almost no colonies formed with *S. aureus* and significantly fewer colonies noted with *E. coli*, the membrane has proved to have excellent antibacterial activity against common oral pathogens. With bio-glass as the only bone graft material depicting inherent antibacterial properties due to the pH-modulating ability and osmotic pressure differences, the addition of selenium could have an added synergistic effect. The mechanism of action of antibacterial activity due to selenium may be attributed to several factors such as the disruption of bacterial cell walls, inhibition of protein, and deoxyribonucleic acid synthesis [20]. Its antioxidant property could also play a crucial role in the depicted antibacterial property. Thus, the combined antioxidant, anti-inflammatory, and antibacterial properties of selenium along with the osteo-inductive properties of bio-glass, collagen, and gelatin may prove to be of great significance. A study exploring the role of zinc bio-glass composite membrane showed improved cell viability and alkaline phosphatase activity with adequate antibacterial activity that did not improve with the addition of zinc [21]. This is in contrast to our results with the selenium bio-glass composite membrane, thus signifying the role of selenium in the antibacterial property of the membrane.

Further, the toxicity analysis with the development of zebrafish embryos revealed that the GBR membrane is highly biocompatible with no adverse effects on embryo development. The rate of the growth of embryos was also not affected when compared to the control. Hence, the material characterization reveals a well-developed GBR membrane that has good surface, chemical, biocompatible, and antibacterial properties ideal for bone regeneration.

Studies have also shown the immunomodulatory role of such collagen-based GBR membranes on bone metabolism [22]. Bio-glass coated collagen membrane has been proven to successfully tune the membrane-mediated osteo-immune response by modulating the expression of various osteogenic factors and inflammatory cytokines. It enhances the expression of osteo-promotive genes leading to the differentiation of bone marrow-derived mesenchymal stem cells into osteoblasts. Various other studies on bio-glass composite membranes have shown that bio-glass enhances osteogenic ability by activating osteogenic genes like the alkaline phosphatase gene, Runt-related transcription factor 2, and the Osteopontin gene [19,23,24]. However, the concentration of the bio-glass also plays a crucial role in the regenerative outcomes.

The present study has a few limitations too. The developed GBR membrane was not assessed for the mechanical characteristics, behavior in biologic fluids, degradation rate, cell attachment, bone mineralization assay, or gene expression pattern using gingival fibroblast and osteoblast cell lines. Antibacterial studies with common periodontal pathogens could have been assessed. The above-mentioned analysis was not performed as the present study aimed at the successful development and characterization of the membrane using preliminary analysis. The novelty of this study remains in the fact that, though various generations of GBR membranes have been developed, the bone regenerative potential of this unique combination of selenium bio-glass, collagen, and gelatin has not been explored yet. With the increased need for fixed dental prostheses in the form of dental implants, the long-term success of implants with improved aesthetics is the need of the hour. GBR with novel bioactive composite scaffold would greatly contribute to the successful regenerative outcomes around implants. Furthermore, in vitro and in vivo studies with animal and human participants should be carried out to analyze the behavior of the developed GBR membrane.

## Conclusions

In conclusion, the developed novel selenium-doped bio-glass-collagen-gelatin composite scaffold has shown excellent surface, chemical, biocompatible, and antibacterial properties that would promote GBR. Further in vitro and in vivo studies should be conducted to further explore the efficacy of this membrane in the treatment of bony defects. 
